# Diversity of *Vibrio navarrensis* Revealed by Genomic Comparison: Veterinary Isolates Are Related to Strains Associated with Human Illness and Sewage Isolates While Seawater Strains Are More Distant

**DOI:** 10.3389/fmicb.2017.01717

**Published:** 2017-09-06

**Authors:** Keike Schwartz, Cindy Kukuc, Nadja Bier, Karin Taureck, Jens A. Hammerl, Eckhard Strauch

**Affiliations:** ^1^Department of Biological Safety, German Federal Institute for Risk Assessment Berlin, Germany; ^2^Landesuntersuchungsanstalt für das Gesundheits- und Veterinärwesen Sachsen Dresden, Germany

**Keywords:** *Vibrio* spp., pathogen, genome, diversity, whole genome sequencing, virulence-associated factors

## Abstract

Strains of *Vibrio navarrensis* are present in aquatic environments like seawater, rivers, and sewage. Recently, strains of this species were identified in human clinical specimens. In this study, *V. navarrensis* strains isolated from livestock in Germany were characterized that were found in aborted fetuses and/or placentas after miscarriages. The veterinary strains were analyzed using phenotypical and genotypical methods and compared to isolates from marine environments of the Baltic Sea and North Sea. The investigated phenotypical traits were similar in all German strains. Whole genome sequencing (WGS) was used to evaluate a phylogenetic relationship by performing a single nucleotide polymorphism (SNP) analysis. For the SNP analysis, WGS data of two American human pathogenic strains and two Spanish environmental isolates from sewage were included. A phylogenetic analysis of concatenated sequences of five protein-coding housekeeping genes (*gyrB, pyrH, recA, atpA*, and *rpoB*), was additionally performed. Both phylogenetic analyses reveal a greater distance of the environmental seawater strains to the other strains. The phylogenetic tree constructed from concatenated sequences of housekeeping genes places veterinary, human pathogenic and Spanish sewage strains into one cluster. Presence and absence of virulence-associated genes were investigated based on WGS data and confirmed by PCR. However, this analysis showed no clear pattern for the potentially pathogenic strains. The detection of *V. navarrensis* in human clinical specimens strongly suggests that this species should be regarded as a potential human pathogen. The identification of *V. navarrensis* strains in domestic animals implicates a zoonotic potential of this species. This could indicate a potential threat for humans, as according to the “One Health” concept, human, animal, and environmental health are linked. Future studies are necessary to search for reservoirs of these bacteria in the environment and/or in living organisms.

## Introduction

*Vibrio navarrensis* was first described as a species isolated from sewage and river water in the Spanish province Navarra in 1991 (Urdaci et al., 1991). Later, some strains from the Baltic Sea were reported that differed in some biochemical reactions to the Spanish strains. However, DNA-DNA hybridization and fatty acid analysis revealed them as *V. navarrensis* and they were classified as *V. navarrensis* biotype *pommerensis* (Jores et al., 2007). All *V. navarrensis* strains showed hemolytic activity on blood agar containing different types of erythrocytes, e.g., human, sheep, horse or cattle blood cells (Jores et al., 2003, 2007). The strains were regarded as environmental strains and found during surveys to determine the occurrence and distribution of pathogenic *Vibrio* species like *Vibrio cholerae* (Urdaci et al., 1991) and *Vibrio vulnificus* (Jores et al., 2007) in aquatic environments.

In 2014, the characterization of *V. navarrensis* isolates associated with human illness was reported in a publication of the Centers for Disease Control and Prevention (CDC), Atlanta, Georgia (Gladney and Tarr, 2014). Most of the strains had been in the CDC strain collection for some time and could not be characterized to the species level by phenotypical methods at the time of isolation. By applying multilocus sequence analysis (MLSA), the strains could be placed in a phylogenetic framework and were assigned to the species *V. navarrensis* (Gladney and Tarr, 2014).

Our laboratory received *Vibrio* strains from a veterinary lab in Saxony, Germany, collected between 1990 and 2011 which contained strains that had been isolated from domestic animals like pig and cattle after abortions. The isolates had been found in animals intended for food production in farms of the German state Saxony that does not border on marine environments. The strains were recovered after abortions from placentas and aborted fetuses (Stephan et al., 2002; Schirmeister et al., 2014). At first, some of these strains had been classified as *V. vulnificus* by phenotypic characterization or were assigned *Vibrio* spp., but could be assigned to *V. navarrensis* by sequencing of the *rpoB* gene coding for the ß-subunit of RNA polymerase (Tarr et al., 2007; Adékambi et al., 2009; Dieckmann et al., 2010). As the animal source of these strains is an unusual source of *Vibrio* bacteria, we compared them to environmental *V. navarrensis* strains of the North Sea and Baltic Sea by studying genotypic and phenotypic traits to find out if the veterinary strains may originate from this environment. Additionally, by including published whole genome sequences of two human pathogenic strains (Gladney et al., 2014) the aim of the study was also to find out if the veterinary isolates are related to these strains which could indicate a pathogenic potential.

The occurrence of *V. navarrensis* strains in freshwater and seawater as well as the isolation from humans and domestic animals reveals a broad ecological range of habitats, which may show a wide genetic diversity of the species. For this purpose, WGS data and sequences of housekeeping genes were applied for constructing phylogenetic trees. For the analyses, whole genome sequencing (WGS) data of four published genomes of *V. navarrensis* strains consisting of two human pathogenic strains from the U.S. and two environmental strains from Spain were included. A number of genes associated with virulence in other human pathogens were found in the *V. navarrensis* genome sequence (Gladney et al., 2014). Presence or absence of some of these virulence-associated genes were investigated by genome comparison and confirmed by PCR analyses.

## Materials and methods

### Bacterial strains

In total, 19 *V. navarrensis* isolates from German sources and one reference strain (CIP 103381 from Spain, isolate from sewage) were investigated in this study (Table 1). Ten strains were obtained from an official veterinary laboratory in Saxony (Landesuntersuchungsanstalt für das Gesundheits- und Veterinärwesen, Dresden) and were mostly isolated from domestic animals after abortions. The veterinary strains were recovered from aborted fetuses or placentas or both. Two environmental seawater strains of the year 2011 were obtained from the Alfred Wegener Institute, Heligoland, and three environmental strains of the year 2015 from a university hospital (Medizinaluntersuchungsamt und Hygiene, Universitätsklinikum Schleswig-Holstein). *Vibrio navarrensis* biotype *pommerensis* strains came from the strain collection of the German Federal Institute for Risk Assessment (BfR). One environmental strain was isolated from a blue mussel harvested in the Wadden Sea of the North Sea.

**Table 1:** *Vibrio navarrensis* strains used in this study.

**Strain**	**Year of isolation**	**Source**	**Origin**
CIP 103381^*^	1982	Reference strain/sewage	Spain
CH-271^**^	1996	Seawater	Baltic Sea
CH-280^**^	1996	Seawater	Baltic Sea
CH-291^**^	1996	Seawater	Baltic Sea
VN-0392	1999	Cattle/placenta	Saxony
VN-0413	2000	Cattle/placenta	Saxony
VN-0414	2000	Cattle/placenta	Saxony
VN-0415	2009	Cattle/fetus	Saxony
VN-0506	2000	Cattle/placenta	Saxony
VN-0507	2000	Cattle/placenta	Saxony
VN-0508	2000	Pig/placenta	Saxony
VN-0509	2001	Pig/fetus	Saxony
VN-0514	2007	Pig/placenta	Saxony
VN-0515	2007	Pig/placenta	Saxony
VN-0516	2015	Brackish water	Schleswig-Holstein
VN-0517	2015	Seawater	Schleswig-Holstein
VN-0518	2015	Seawater	Schleswig-Holstein
VN-0519	2011	Blue mussel	Lower Saxony
VN-3125	2011	Seawater	Kattegat
VN-3139	2011	Seawater	Kattegat

* *Identical to ATCC 51183*.

** *Strains of V. navarrensis biotype pommerensis. Strain CH-291 was deposited as DSM 15800*.

### Biochemical characterization

*Vibrio navarrensis* strains were routinely cultivated in LB medium (Merck KGaA, Darmstadt, Germany) at 37°C. Strains were characterized by biochemical tests used in routine diagnostics of the National Reference Laboratory (NRL) for Monitoring Bacteriological Contamination of Bivalve Mollusks located at the BfR. Tests included growth in 1% peptone water with 0, 3, 8, and 10% NaCl, cytochrome oxidase, sensitivity to the vibriostatic agent O/129 (10 and 150 μg), lysine decarboxylase, arginine dihydrolase, ornithine decarboxylase, nitrate reductase (all supplemented with 1% NaCl), and utilization of a number of carbohydrates (Farmer et al., 2003). Phenylalanine deamination was tested on phenylalanine agar IDM 31 (Mast Diagnostica GmbH, Reinfeld, Germany) supplemented with 1% NaCl. To ensure test results, the following Gram-negative bacterial strains served as controls: *Aeromonas hydrophila* ATCC 7966 (positive control for cytochrome oxidase test, resistance to O/129), *Escherichia coli* DSM 1103 (positive control for oxidative acid production from D-glucose, maltose, D-mannose, and trehalose; negative control for phenylalanine deaminase test, urease test, and citrate degradation test), *Klebsiella oxytoca* DSM 25736 (positive control for oxidative acid production from adonitol, L-arabinose, cellobiose, dulcitol, *myo*-inositol, lactose, D-mannitol, melibiose, raffinose, L-rhamnose, salicin, D-sorbitol, L-sorbose, sucrose, and D-xylose, esculin and citrate degradation tests), *Morganella morganii* DSM 30117 (negative control for oxidative acid production from cellobiose, trehalose, and D-xylose), *Proteus mirabilis* DSM 4479 (positive control for phenylalanine deaminase test, urease test, and H_2_S production test; negative control for cytochrome oxidase test, oxidative acid production from adonitol, L-arabinose, dulcitol, *myo*-inositol, lactose, D-mannitol, D-mannose, melibiose, raffinose, L-rhamnose, salicin, D-sorbitol, L-sorbose, and sucrose, esculin degradation test), and *Shigella sonnei* DSM 25715 (negative control for oxidative acid production from D-glucose and maltose, H_2_S production test). According to quality control standards of the NRL, functionality of liquid growth media supplemented with 1% NaCl was tested with the following *Vibrio* spp. strains: *V*. *alginolyticus* DSM 2171 (positive control for growth in 1% peptone water with 3, 6, and 8% NaCl), *V. cholerae* DSM 101014 (positive control for nitrate reductase test, lysine decarboxylase test, ornithine decarboxylase test, indole production test, growth in 1% peptone water with 0% NaCl; negative control for arginine dihydrolase test), *V. cholerae* ATCC 14035 (susceptibility to O/129), *V. furnissii* DSM 14383 (positive control for arginine dihydrolase test; negative control for lysine decarboxylase and ornithine decarboxylase tests), *V. metschnikovii* LMG 4416 (positive control for acetoin production test; negative control for nitrate reductase test, indole production test), and *V. parahaemolyticus* DSM 101031 (negative control for acetoin production test). Biochemical testing was repeated twice.

### Hemolytic activity tests

Blood agar plates were prepared with Mueller-Hinton agar (Oxoid GmbH, Wesel, Germany) supplemented with 1% NaCl and contained 4% sheep (BfR, Berlin, Germany) or 4% human erythrocytes (German Red Cross, blood donation service, Berlin-Wannsee, Germany). Erythrocytes were washed three times in cold phosphate buffered saline and pelleted for 5 min at 400 × g and 10°C before use. Prior to hemolysis assay, bacteria were cultivated from glycerol stocks on Mueller-Hinton agar plates overnight at 37°C. Four milliliters of Mueller-Hinton broth were inoculated with one single colony and incubated for 12–14 h at 37°C with constant shaking (200 rpm). All culture media were supplemented with 1% NaCl. In order to investigate the hemolytic activity of the strains, 10 μl of the overnight cultures were spotted on a blood agar plate and incubated at 37°C to obtain macrocolonies. Zones of hemolysis around the macrocolonies were visually controlled and scored for up to 72 h. All experiments were performed twice. Strains which did not reveal hemolysis zones on sheep blood were recultivated from glycerol stocks, passaged three times on sheep blood agar plates consisting of Special Blood Agar Base DM101 (Mast Diagnostica GmbH, Reinfeld, Germany) supplemented with 5% defibrinated sheep blood (BfR, Berlin, Germany) and retested on the modified agar plates as described above.

### *rpoB* sequencing

Bacterial strains were grown overnight and genomic DNA was extracted using the RTP Bacteria DNA Mini Kit from Stratec Molecular, Berlin, Germany, according to the manufacturer's instructions. Analyses of the *rpoB* gene were performed using the PCR primers and sequencing primers described earlier (Mollet et al., 1997; Tarr et al., 2007; Table S1). Briefly, a 984 bp fragment of the *rpoB* coding sequence was amplified using the primers CM32b and 1110F. For sequencing of the amplification products, the PCR primers and two additional primers (1661F, 1783R) were used. PCR conditions were according to the protocol given in Tarr et al. (2007).

### Whole genome sequencing (WGS) and single nucleotide polymorphism (SNP) analysis

Genomic DNA of *V. navarrensis* isolates was prepared using the PureLink Genomic DNA Mini Kit (Invitrogen, Karlsruhe, Germany). DNA libraries were generated with the Nextera XT DNA Sample Preparation Kit according to the manufacturer's protocol (Illumina Inc., San Diego, CA, USA). DNA sequencing using the MiSeq Reagent v3 600-cycle Kit (2 × 300 cycles) was performed on the MiSeq benchtop (Illumina Inc., San Diego, CA, USA). For *de novo* assembling of raw sequencing reads, the SPAdes (version 3.5.0) algorithm was used. Initial annotation of the genomes was performed by using the automated Prokaryotic Genome Annotation Pipeline of the NCBI website. Further genetic features and elements of the genomes were identified using the Bacterial Analysis Pipeline and the Phage Search Tool (Zhou et al., 2011). Putative prophage sequences were recorded based on clusters of more than six phage-like genes within a sequence region of the analyzed genome. Therefore, phage-like genes were identified according to their similarity against sequences of the NCBI database. Additionally, the genome annotation of the isolates was analyzed for phage specific terms like “protease,” “integrase,” and “tail fiber.” Predicted prophage regions were assessed according to the recommendations (Zhou et al., 2011). Initial plasmid prediction was performed by using *de novo* assemblies of genomes with the web-based tool “PlasmidFinder” of the Center for Genomic Epidemiology (Carattoli et al., 2014). Furthermore, contigs with significantly higher sequence coverage than the rest of the genomic contigs were applied to the BLASTN search of the NCBI database and screened for similarities to known plasmids.

The SNP tree was conducted by using CSI Phylogeny 1.4 (Center for Genomic Epidemiology) under default settings and the exclusion of heterozygous SNPs. To identify SNPs, all input sequences were mapped to the *V. navarrensis* 0053-83 genome as reference (JMCF01000001) and screened for relevant nucleotide variations as previously described (Kaas et al., 2014). The following criteria for high quality SNP calling and filtering were chosen: (I) a minimum depth of 10 × at SNP positions, (II) a min. relative depth of 10% at SNP positions, (III) a min. distance of 10 bp between SNPs, (IV) a min. SNP quality of 30, (V) a min. read mapping quality of 25, and (VI) a min. *Z*-score of 1.96. Site validation for each SNP position was performed. SNPs that fail the necessary requirements were excluded in the final analysis. Based on concatenated alignments of high quality SNPs, maximum likelihood trees were created using FastTree version 2.1.7 (Price et al., 2010).

The concatenated sequences derived from whole genomes were used for screening on virulence-associated genes using the BLASTN algorithm of the NCBI database (https://www.ncbi.nlm.nih.gov). The web-based tool was used with standard settings.

### Multilocus sequence analysis (MLSA) of housekeeping genes

Bacterial strains were grown overnight and genomic DNA was extracted as described above. MLSA was performed on four protein-coding housekeeping genes making use of the *Vibrio* spp. MLSA website (https://pubmlst.org/vibrio/info/Vibrio_primers.pdf) developed by Keith Jolley and sited at the University of Oxford (Jolley and Maiden, 2010). The 25 μl PCR mixtures contained 1 × PCR buffer (2 mM MgCl_2_), 0.2 mM of each dNTP, 0.2 μM of each primer, 1.5 U DreamTaq DNA Polymerase (Thermo Fisher Scientific Biosciences GmbH, St. Leon-Rot, Germany), and 1 μl of genomic DNA. For amplification of *gyrB*, the primers VigyrBF and VigyrBR were used. Amplification of *atpA* was carried out with the primers Vi_atpAdg_F and Vi_atpAdg_R. PCR reactions were performed using a Mastercycler ep gradient (Eppendorf AG, Hamburg, Germany). PCR products were purified using the MSB Spin PCRapace Kit according to the manufacturer's instructions (Stratec Molecular GmbH, Berlin, Germany) and sequenced (Eurofins Genomics GmbH, Ebersberg, Germany). The sequences were assembled and analyzed using the Lasergene software SeqMan Pro version 12.0 (DNASTAR Inc., Madison, WI, USA) and the software Accelrys Gene version 2.5 (Accelrys Inc., San Diego, CA, USA). Allele sequences including the *rpoB* sequences were concatenated in the order of loci *gyrB*-*pyrH*-*recA*-*atpA*-*rpoB* to generate a 2,893 bp concatemer for each strain. A phylogenetic tree was constructed with MEGA version 6.0 (Tamura et al., 2013) based on the alignment of the concatenated allele sequences using the neighbor-joining method with the Kimura 2-parameter model. Bootstrapping with 1,000 replications was performed to verify the robustness of the tree.

### PCR typing of virulence-associated genes

Extraction of genomic DNA and PCR reactions were performed as described above with 5 ng of template DNA. PCR primers, target genes, and amplicon sizes are shown in Table S1. Primer sequences were derived from whole genome contigs of three *V. navarrensis* strains (Gladney et al., 2014) using the software Accelrys Gene version 2.5. Accession numbers of contigs are given in Table S1. The PCR running conditions were as follows: an initial denaturation step at 94°C for 4 min, 30 cycles of denaturation at 94°C for 15 s, primer annealing at 55°C for 30 s and extension at 72°C for 45 s, and a final extension step at 72°C for 7 min. PCR products were analyzed in agarose gels to determine the product lengths. Selected PCR products were sequenced for confirmation.

### Accession numbers

**Figure 1:**
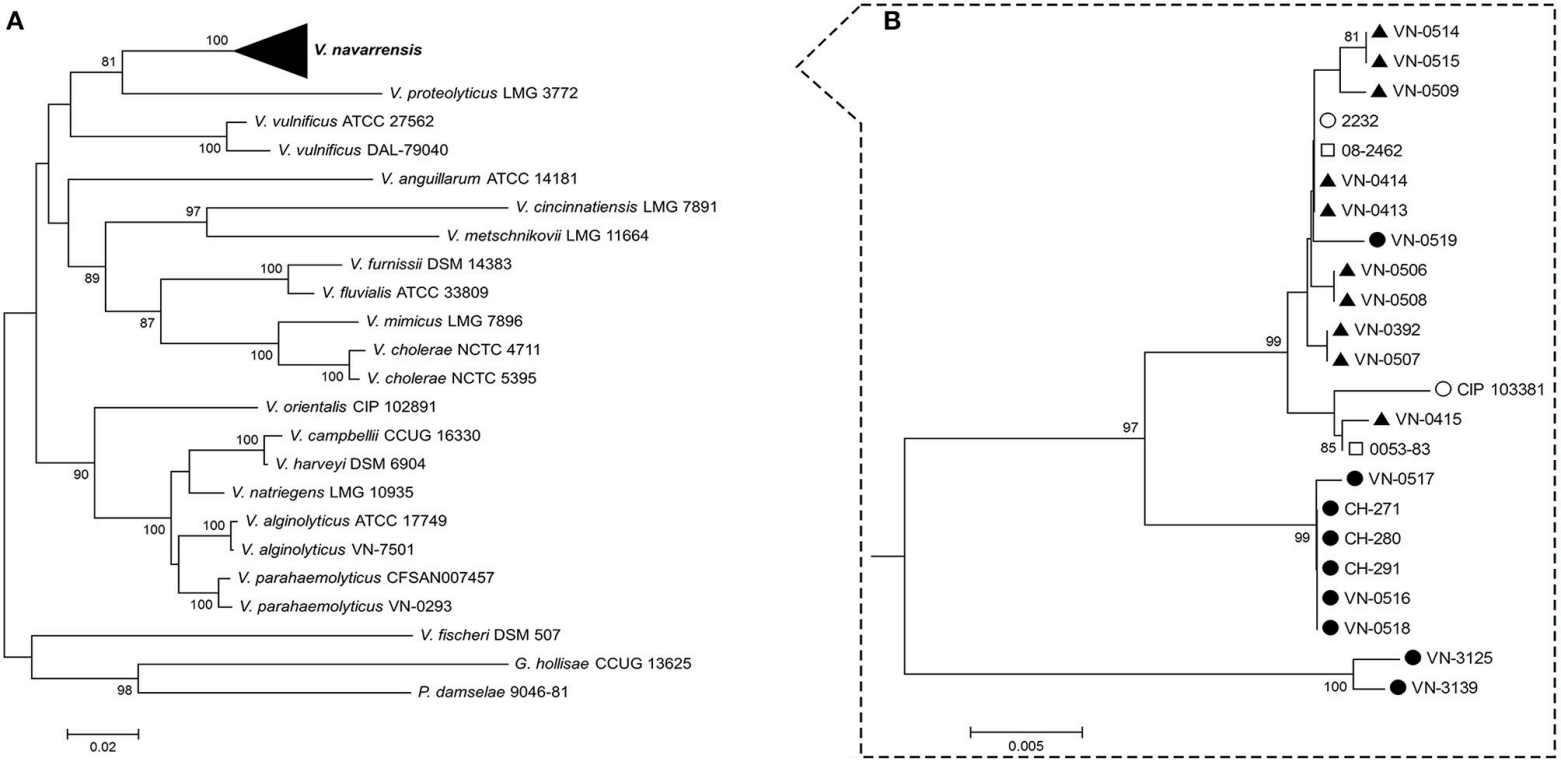
Phylogenetic tree of different *Vibrionaceae* strains **(A)** and subtree of *V. navarrensis* strains **(B)** based on partial *rpoB* sequences (871 bp). The evolutionary history was inferred using the neighbor-joining method with Kimura 2-parameter distance model in MEGA version 6.0. Bootstrap values above 75% are shown next to the nodes (*n* = 1,000 replicates). Scale bars represent base substitutions per site. German veterinary (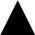) and environmental (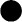), Spanish environmental (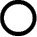), and human pathogenic *V. navarrensis* strains (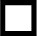; from the U.S.) (Gladney and Tarr, 2014) form a distinct cluster within a number of different *Vibrionaceae* strains. *rpoB* sequences of non-*V. navarrensis* strains and *V. navarrensis* strains 2232, 08-2462, CIP 103381 (identical to ATCC 51183), 0053-83, VN-3125, and VN-3139 were obtained from GenBank at National Center for Biotechnology Information (NCBI). Accession numbers are given in Materials and Methods.

Nucleotide sequences were deposited in the European Nucleotide Archive (ENA) with the following accession numbers: sequences of partial *rpoB* gene accession numbers LT546547-LT546563 (*rpoB* sequences of three strains already in database, see Figure 1), sequences of partial *atpA* gene accession LT546564-LT546583, sequences of partial *gyrB* gene accession LT546584-LT546603, sequences of partial *pyrH* gene accession LT546604-LT546623, and sequences of partial *recA* gene accession LT546624-LT546643.

Genome sequences of *V. navarrensis* isolates have been deposited in GenBank at National Center for Biotechnology Information (NCBI) under the accession numbers MPKB00000000 to MPKU00000000 (see **Table 3**).

For *rpoB* phylogeny presented in Figure 1, *rpoB* sequences of non-*V. navarrensis* strains and *V. navarrensis* strains 2232, 08-2462, CIP 103381 (identical to ATCC 51183), 0053-83, VN-3125, and VN-3139 were obtained from GenBank at NCBI. Accession numbers are FN423814 (LMG 3772), FN423805 (ATCC 27562), LMYA01000047 (DAL-79040), MCJC01000044 (ATCC 14181), FN423808 (LMG 7891), FN423806 (LMG 11664), HG794494 (DSM 14383), CP014035 (ATCC 33809), FN423804 (LMG 7896), FN423803 (NCTC 4711), CP013317 (NCTC 5395), AFWH01000033 (CIP 102891), FN423816 (CCUG 16330), FN423810 (DSM 6904), FN423812 (LMG 10935), FN423802 (ATCC 17749), LVYF01000041 (VN-7501), JNUL02000012 (CFSAN007457), MVKN01000048 (VN-0293), FN423813 (DSM 507), FN423801 (CCUG 13625), EF064429 (9046-81), JMCH01000016 (2232), JMCI01000045 (08-2462), JMCG01000002 (ATCC 51183), JMCF01000001 (0053-83), KJ647757 (VN-3125), and KJ647770 (VN-3139).

## Results and discussion

### *rpoB* phylogeny

Determination of partial *rpoB* sequences has proved a reliable method for species identification for bacteria of the family *Vibrionaceae* (Tarr et al., 2007; Adékambi et al., 2009; Dieckmann et al., 2010). The sequences of an 871 bp internal fragment of the coding sequence of the *rpoB* gene were identified for all isolates of this study. *rpoB* sequences of two Spanish environmental *V. navarrensis* strains [CIP 103381 (identical to ATCC 51183) and 2232] and two human pathogenic strains (0053-83 and 08-2462; Gladney et al., 2014) available in public databases were included for the construction of a phylogenetic tree (Figure 1B). All strains fell into a cluster that formed a distinct species among a number of different *Vibrio* species (Figure 1).

With the exception of two strains, the identity of the sequences of most *V. navarrensis* strains was greater than 98% in the sequenced region of 871 bp. In a previous study, we observed that in many *Vibrio* spp. the lowest sequence identity (determined by ClustalW) of this gene fragment was around 98% on species level (Dieckmann et al., 2010). The identity of the *rpoB* sequences of two *V. navarrensis* strains from seawater (VN-3125 and VN-3139) to the *rpoB* sequences of the remaining *V. navarrensis* strains was only ca. 96%. Only in three of the 40 polymorphic sites of the sequenced fragment, nonsynonymous substitutions leading to amino acid exchanges in the gene product were discovered. Two identical amino acid exchanges were observed only in the more distantly related strains VN-3125 and VN-3139.

The *rpoB* tree showed that the veterinary isolates from domestic animals clustered with two Spanish environmental strains [CIP 103381 (identical to ATCC 51183) and 2232] and two human pathogenic strains (Gladney et al., 2014; Figure 1B). Also one environmental strain, VN-0519 isolated from a blue mussel harvested from a mussel production area, fell into this cluster, while six environmental seawater isolates from Germany formed a separate subcluster.

### Phenotypic characteristics

All strains (Table 1) were phenotypically tested using a panel of standard biochemical reactions (Table 2). The biochemical properties were fairly homogenous with more than 90% of the strains showing the same result (only few variable reactions). Comparing to published results, biochemical characteristics were typical as described for *V. navarrensis* (Urdaci et al., 1991; Farmer and Janda, 2004; Jores et al., 2007; Gladney and Tarr, 2014; Farmer et al., 2015). The strains were negative for Voges-Proskauer test, arginine dihydrolase, lysine decarboxylase, and ornithine decarboxylase and were positive for sucrose fermentation and phenylalanine deaminase activity. The three strains of the biovar *pommerensis* (CH-271, CH-280, CH-291) were not studied in further details, as the biovar specific reactions were not part of our standard reaction panel (Jores et al., 2007).

**Table 2:** Phenotypic characterization of *V. navarrensis* strains.

**Phenotypic test**	**No. of isolates (%)**	
	**+**	**−**
Cytochrome oxidase	20 (100)	0 (0)
Nitrate reductase	20 (100)	0 (0)
Arginine dihydrolase	0 (0)	20 (100)
Lysine decarboxylase	0 (0)	20 (100)
Ornithine decarboxylase	0 (0)	20 (100)
Phenylalanine deaminase	18 (90)	2 (10)
Urease	0 (0)	20 (100)
**Production of:**		
Acetoin (Voges-Proskauer reaction)	0 (0)	20 (100)
H_2_S	0 (0)	20 (100)
Indole	20 (100)	0 (0)
**Oxidative acid production from:**		
Adonitol	0 (0)	20 (100)
L-Arabinose	1 (5)	19 (95)
Cellobiose	19 (95)	1 (5)
Dulcitol	0 (0)	20 (100)
D-Glucose	20 (100)	0 (0)
*myo*-Inositol	0 (0)	20 (100)
Lactose	8 (40)	12 (60)
Maltose	20 (100)	0 (0)
D-Mannitol	20 (100)	0 (0)
D-Mannose	18 (90)	2 (10)
Melibiose	3 (15)	17 (85)
Raffinose	0 (0)	20 (100)
L-Rhamnose	2 (10)	18 (90)
Salicin	1 (5)	19 (95)
D-Sorbitol	0 (0)	20 (100)
L-Sorbose	0 (0)	20 (100)
Sucrose	20 (100)	0 (0)
Trehalose	20 (100)	0 (0)
D-Xylose	0 (0)	20 (100)
**Degradation of:**		
Esculin	4 (20)	16 (80)
Citrate (Simmons citrate reaction)	5 (25)	15 (75)
**Growth in 1% peptone water**		
+ 0% NaCl	0 (0)	20 (100)
+ 3% NaCl	20 (100)	0 (0)
+ 8% NaCl	0 (0)	20 (100)
+ 10% NaCl	0 (0)	20 (100)
Susceptibility to O/129 (10 μg/150 μg)	20 (100)	0 (0)
**Hemolysis of:**		
Human erythrocytes	19 (95)	1 (5)
Sheep erythrocytes	19 (95)	1 (5)

Since the veterinary laboratory in Saxony, from which the strains were obtained, did not use molecular diagnostic techniques such as PCR and DNA sequencing, the isolates from animals had been phenotypically determined either as *Vibrio* sp. or as *V. vulnificus*. Misidentification by traditional methods happened, as phenotypic characteristics of *V. navarrensis* and *V. vulnificus* are very similar (Gladney and Tarr, 2014). The phenotypic characterization of all 20 strains was also done to find out if distinct biochemical properties could be correlated to the source of the strains. The results of these investigations, however, did not reveal significant differences between the strains (see Table 2).

As *V. navarrensis* strains show hemolytic activity (Jores et al., 2003, 2007), we investigated all strains on agar plates containing sheep erythrocytes or human erythrocytes. Most strains did not show hemolytic activity within 24 h. However, after incubation for up to 72 h all strains but one were hemolytic against human erythrocytes. On sheep blood agar, six strains (two environmental and four veterinary strains) did not show hemolysis at first. However, after modification of the assay medium and repeated streaking on sheep blood agar also these strains (except one) displayed hemolysis zones. With sheep and human erythrocytes, hemolysis zones surrounding colonies were clear indicating a ß-hemolysis with complete degradation of hemoglobin (Zhang and Austin, 2005).

### Whole genome sequencing

The results of genomes of ten German isolates (five veterinary and five environmental strains) are shown in Table 3. The genome sizes range from 4.14 to 4.90 Mbp and the GC contents of the genomes vary between 47.5 and 48.1%. The predicted number of coding sequences range from 3,559 to 4,247. The published genomes of two Spanish strains (ATCC 51183 and 2232) and two human pathogenic strains (0053-83 and 08-2462) vary between 4.2 and 4.4 Mbp (Gladney and Tarr, 2014). It was noted that the genomes of all five veterinary strains are also in this range, while the genomes of the five isolates from marine environments including the mussel isolate are larger (4.6–4.9 Mbp, Table 3). It is possible that the greater genome sizes of marine strains reflect a wider range of metabolic capabilities compared to the veterinary and human pathogenic strains. Some bacteria, especially those adapted to specific niches (e.g., pathogenic strains adapted to specific host environments) can lose metabolic capabilities leading to a reduction in genome size (Raskin et al., 2006). Further studies may address this question.

**Table 3:** Results of the whole genome sequence analysis of veterinary and environmental *V. navarrensis* strains.

**Feature**	**CH-280**	**VN-0392**	**VN-0415**	**VN-0507**	**VN-0509**	**VN-0514**	**VN-0516**	**VN-0518**	**VN-0519**	**VN-3125**
**Genome size** (bp)	4,899,705	4,287,414	4,138,545	4,271,170	4,356,049	4,278,964	4,605,884	4,684,360	4,788,163	4,765,427
**GC content** (%)	47.45	48.02	48.08	48.04	48.09	48.07	47.71	47.75	47.94	47.57
**Genes** (total)^*^	4,440	3,935	3,784	3,857	3,923	3,909	4,162	4,280	4,331	4,338
**CDS** (total)^**^	4,319	3,811	3,661	3,731	3,781	3,779	4,058	4,136	4,201	4,233
CDS (coding)	4,247	3,737	3,559	3,658	3,703	3,693	4,005	4,098	4,149	4,186
**RNA genes** (total)^***^	121	124	123	126	142	130	104	104	130	105
**rRNAs** (5S, 16S, 32S)^***^	7, 8, 7	9, 11, 10	5, 9, 7	7, 8, 7	8, 12, 11	7, 8, 11	4, 4, 4	5, 9, 1	7, 8, 9	8, 7, 7
**tRNAs**	95	90	98	100	107	100	88	85	101	79
**ncRNAs**	4	4	4	4	4	4	4	4	5	4
**Pseudogenes** (total)	72	74	102	73	78	86	53	38	52	47
CRISPR Arrays	2	3	3	1	1	1	1	0	1	0
**Predicted prophages** (no.)	7	3	7	7	2	9	1	2	4	4
intact	n.d.	1	n.d.	n.d.	1	n.d.	1	n.d.	1	3
incomplete	6	1	7	7	1	9	n.d.	1	2	1
questionable	1	1	n.d.	n.d.	n.d.	n.d.	n.d.	1	1	n.d.
**Plasmids**	n.d.	n.d.	n.d.	n.d.	n.d.	n.d.	n.d.	n.d.	n.d.	ColRNAI, 88.39%
**GenBank accession**										
Bioproject	PRJNA353389	PRJNA353302	PRJNA353299	PRJNA353297	PRJNA353295	PRJNA353294	PRJNA353292	PRJNA353290	PRJNA353289	PRJNA353288
Biosample	SAMN06014880	SAMN06013691	SAMN06013689	SAMN06013684	SAMN06013686	SAMN06013681	SAMN06013683	SAMN06013678	SAMN06013679	SAMN06013680
Accession	MPKT00000000	MPKB00000000	MPKE00000000	MPKG00000000	MPKI00000000	MPKJ00000000	MPKL00000000	MPKN00000000	MPKO00000000	MPKP00000000

* *Nucleotide sequences from the start codon (ATG) to the stop codon*.

** *Nucleotide sequence that is translated to form proteins*.

*** *Including partial sequences of the respective element*.

*n.d., not detected*.

As bacteriophages are involved in horizontal gene transfer, the WGS data were analyzed with Phage Search Tool (Zhou et al., 2011). The search for phage sequences revealed the occurrence of several prophage sequences as expected (Table S2). Phages are one of the major forces driving horizontal gene transfer (Raskin et al., 2006). Most prophage sequences are related to giant viruses and of lower significance. In some strains, however, prophage sequences possibly encoding intact phages were detected (Enterobacteria phages HK630 and HK629, *Vibrio* phages martha 12B12 and VPUSM 8). However, no information about the phages except the genome sequences are available (Table S2). In two marine strains (VN-0516 and VN-3125), a possibly intact phage was found that is related to *Vibrio* phage VCY Φ. This phage is a small filamentous phage (approximately 7.1 kbp) and was found in association with environmental *V. cholerae* strains in ponds (Xue et al., 2012). Bioinformatics indicated the presence of a plasmid in only one strain (VN-3125). A small region of 638 bp was identified possessing high identity (>99%) to a replication region present in several *Enterobacteriaceae* plasmids [e.g., plasmid p8401 in *E. coli* (accession CP012198)]. In the four published *V. navarrensis* genomes, no plasmid sequences were reported so far.

### SNP phylogeny of whole genome sequences

**Figure 2:**
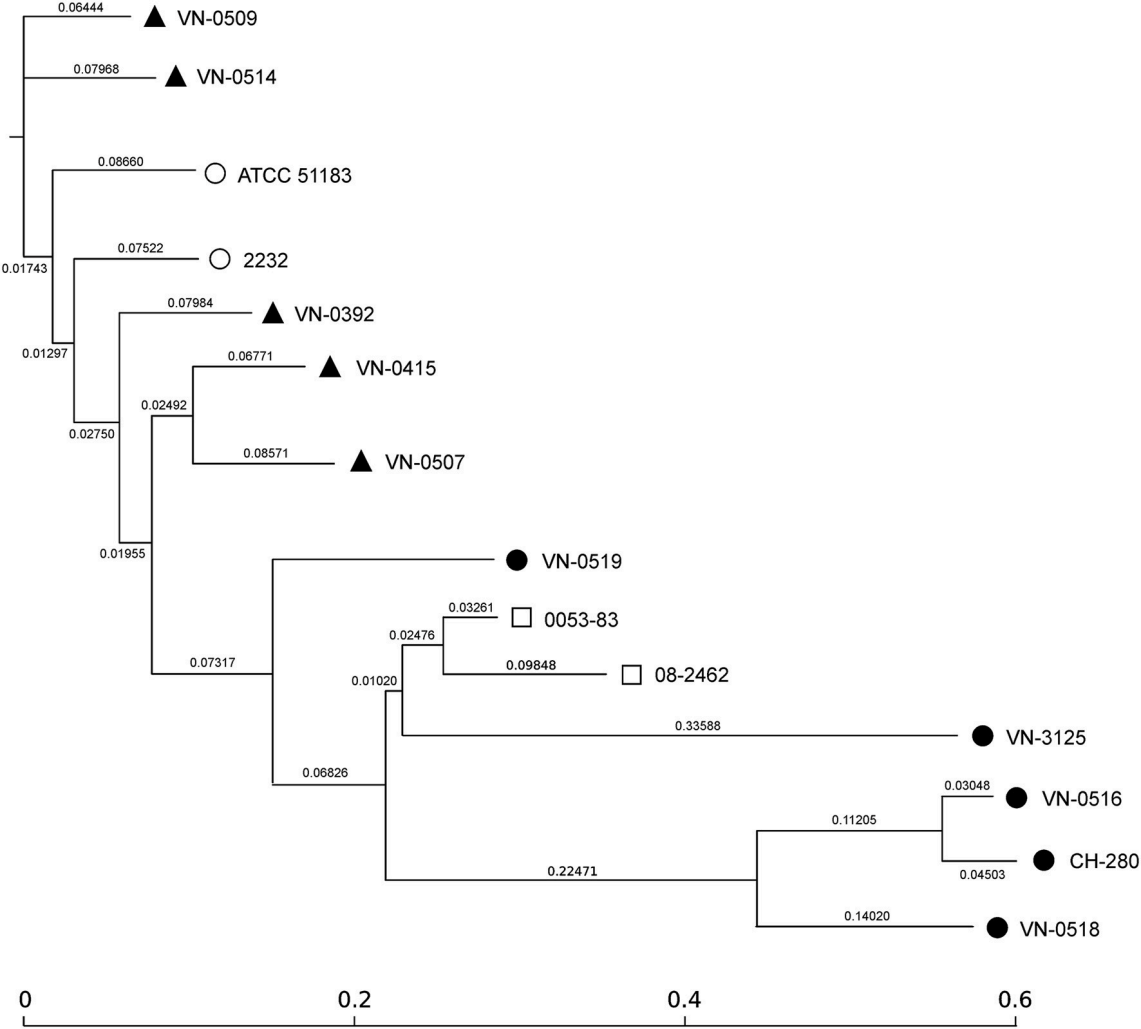
SNP-based phylogeny tree of German veterinary (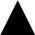) and environmental (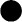) *V. navarrensis* isolates, two human (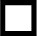) isolates from the U.S., and two Spanish environmental (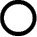) strains [CIP 103381 (identical to ATCC 51183) and 2232 (see text)]. SNP-tree was conducted by using CSI Phylogeny 1.4 under default settings. Single nucleotide polymorphisms (SNPs) were called by mapping to the *V. navarrensis* 0053-83 genome as reference (JMCF01000000). Criteria for high quality SNP calling and filtering are described in Material and Methods. Based on concatenated alignments of high quality SNPs, maximum likelihood trees were created using FastTree version 2.1.7. Scale bar represents the number of nucleotide substitutions per site and numbers indicate branch length.

For an SNP analysis, additional published WGS data of two human pathogenic strains (0053-83 and 08-2462) and two environmental Spanish strains (ATCC 51183 and 2232) were included. The human isolates were from human specimen isolated in the U.S. and the environmental strains were Spanish isolates from sewage (Urdaci et al., 1991). To identify SNPs, all input sequences were mapped to the *V. navarrensis* 0053-83 genome as reference (JMCF01000000) and screened for relevant nucleotide variations (Kaas et al., 2014). In total, the concatenated contigs used for the SNP analysis comprised approx. 3.688 Mbp and the number of SNPs between the strains varied between 10,000 to 63,000 (Figure S1). Using the concatenated alignments of high quality SNPs, maximum likelihood trees were created using FastTree 2 (Price et al., 2010; Figure 2). Based on the length of the branches, the five veterinary strains and the two Spanish environmental strains differ but are related. The two human isolates (0053-83, 08-2462) are closer related to each other (the SNP difference is around 16,000 between the two strains) but are more distant to the veterinary strains and the Spanish strains (approximately 30,000 SNPs). All marine isolates from Germany with the exception of the mussel strain VN-0519 are clearly separated from the other strains, but differ also from each other (SNP differences between 30,000 and 63,000). Only the seawater strains VN-0516 and CH-280 are closer related (difference approximately 10,000 SNPs) which indicates that VN-0516 may belong to the subspecies *V. navarrensis* biotype *pommerensis*.

The SNP analysis revealed clearly that the seawater strains are distant to the remaining *V. navarrensis* strains. In *E. coli*, SNP analysis of the core genome consisting of 1,429 genes revealed 128,214 variable sites for a pathovar (Von Mentzer et al., 2014) and the average SNP differences between two related clades of isolates from different hosts were below 100 within a clade and below 1,800 between two clades (Schaufler et al., 2016). The SNP calculation for the *V. navarrensis* strains (without the seawater strains) vary from 18,000 to 32,000 variable sites. Thus, a close relatedness of all strains cannot be deduced from SNP data (Figure S1).

### Phylogenetic relationship from multilocus sequence analysis of housekeeping genes

We applied the *Vibrio*-MLSA scheme available on the PubMLST website and included sequences from published WGS data of the four *V. navarrensis* strains (Gladney et al., 2014) into the phylogenetic analysis. To increase the depth of the analysis, the *rpoB* sequences were added and the sequences of the concatemer were arranged in the order *gyrB*-*pyrH*-*recA*-*atpA*-*rpoB*. Concatemers of sequences of housekeeping genes were created, as comparison of housekeeping genes is used for infra-species resolution and determination of the clonality of strains (Glaeser and Kämpfer, 2015).

**Figure 3:**
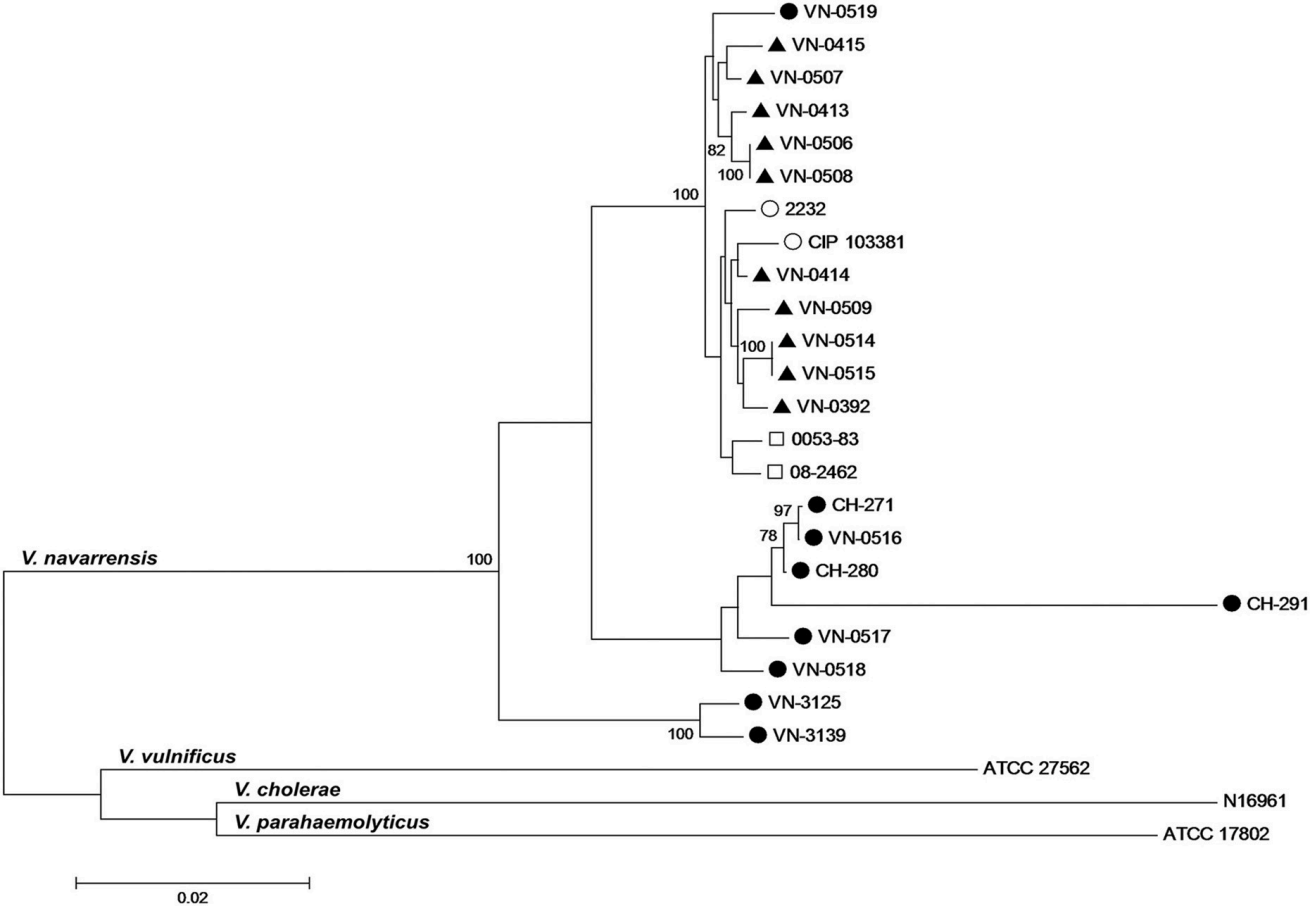
Phylogenetic relationship of German veterinary (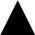) and environmental (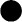) *V. navarrensis* isolates, human (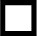) isolates from the U.S., and Spanish environmental (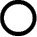) strains based on concatenated sequences of five protein-coding housekeeping genes (*gyrB*-*pyrH*-*recA*-*atpA*-*rpoB*; 2,893 bp). Sequences of *V. navarrensis* 0053-83, 08-2462, CIP 103381 (identical to ATCC 51183), and 2232 were obtained from previous studies (Gladney and Tarr, 2014; Gladney et al., 2014). Sequences of *V. vulnificus* ATCC 27562, *V. cholerae* N16961, and *V. parahaemolyticus* ATCC 17802 from *Vibrio* spp. MLSA website (http://pubmlst.org/vibrio/) and GenBank at NCBI, respectively, were included for comparison. The evolutionary history was inferred using the neighbor-joining method with Kimura 2-parameter distance model in MEGA version 6.0. Bootstrap values above 75% are shown next to the nodes (*n* = 1,000 replicates). Scale bar represents 0.02 base substitutions per site.

The phylogenetic analysis of the concatemers displayed one subcluster containing the veterinary isolates, the mussel isolate, and the reference strain ATCC 51183 (identical to CIP 103381; Figure 3). The sewage strain 2232 and the two human isolates were also placed in this cluster. It should be noted that the two Spanish environmental strains were isolated from sewage in towns of the Spanish province Navarra away from the coast (Urdaci et al., 1991). The concatemers of the German seawater isolates split into three branches and were clearly distant to the subcluster formed by the remaining strains. The MLSA tree indicates a stronger relationship between the veterinary strains, the Spanish strains and the human pathogenic strains than the SNP phylogenetic tree. This discrepancy is likely to be explained by the fact that the housekeeping genes are encoding essential cellular functions and are therefore more conserved and evolve relatively slowly (Hanage et al., 2006).

The observation that the environmental Spanish strains are related to the veterinary strains is a remarkable observation. The Spanish strains were isolated from low salinity aquatic environments away from the coast (Urdaci et al., 1991) in rivers and sewage. As bacteria of the genus *Vibrio* are regarded as environmental aquatic bacteria, it seems possible that *V. navarrensis* may also occur in freshwater in regions of Germany and that the veterinary isolates were ingested by the animals through uptake of surface water. This hypothesis is supported by the results of the phylogenetic analyses which show that the veterinary strains are distinctly different from the German seawater strains. Unfortunately, there is no knowledge concerning a possible origin of the veterinary strains as respective investigations were not undertaken. The phylogenetic studies also indicate that the human strains from the U.S. are more related to the veterinary ones. However, no further information concerning the American strains is available.

The mussel strain (VN-0519) is the only strain from a marine environment that shows a stronger relationship to the veterinary and human isolates. It should be noted that knowledge on natural habitats of *V. navarrensis* are fragmentary, as only few publications on this species are available. In one report from Thailand, partial sequences of 16S rDNA of uncultured bacteria recovered from the gut of marine shrimps were 99% identical to 16S rDNA sequences of *V. navarrensis* (Rungrassamee et al., 2013). In another recent paper, the occurrence of *V. navarrensis* in larval midgut of the date palm root borer *Oryctes agamemnon* in Saudi-Arabia (El-Sayed and Ibrahim, 2015) was detected based on sequence analysis of the gut microbiome. According to this paper, the endosymbiotic bacterial community was dominated by *Vibrionaceae* revealing that these bacteria can be prevalent in an insect environment.

### Presence/absence of virulence-associated factors

To find out if environmental *V. navarrensis* strains can be distinguished from the veterinary strains and human pathogenic strains, the presence or absence of genes coding for virulence-associated factors were investigated by BLASTN searches of the WGS data and by PCR genotyping of the 20 available strains. A number of putative candidate genes were selected based on the published genomes of the reference strain ATCC 51183 (identical to CIP 103381) and the two human *V. navarrensis* strains 0053-83 and 08-2462. PCR primers were designed for a number of genes using the WGS data of these strains (Table S1). The genes targeted were coding for potential virulence factors with cytolytic or hemolytic activities and parts of secretion systems (Gladney et al., 2014). Table 4 summarizes the results of these investigations for all *V. navarrensis* strains including the four published strains.

**Table 4:** Presence/absence of virulence-associated traits in veterinary, human, and environmental *V. navarrensis* isolates based on WGS data.

**Strain**	**Source code**	Virulence-associated genotypic traits^*^													
		***cps***	**T6SS *DUF877***	**T6SS *DUF770***	**T6SS *vasD***	***pilV***	***pilW***	***tlh***	***osmY***	***vvhA***	***δ-vph***	***hlyD***	hlyIII^**^	rtx^**^	**ORF12**
VN-0392	vet														
VN-0413	vet														
VN-0414	vet														
VN-0415	vet														
VN-0506	vet														
VN-0507	vet														
VN-0508	vet														
VN-0509	vet														
VN-0514	vet														
VN-0515	vet														
08-2462^**^	hum														
0053-83^**^	hum														
CIP 103381^***^	env-Sp														
2232^**^	env-Sp														
CH-271	env-G														
CH-280	env-G														
CH-291	env-G														
VN-0516	env-G														
VN-0517	env-G														
VN-0518	env-G														
VN-0519	env-G														
VN-3125	env-G														
VN-3139	env-G														

*vet, veterinary; hum, human; env-Sp, environmental-Spain; env-G, environmental-Germany*.

* *In WGS analysis, gene sequences of V. navarrensis 08-2462 (cps, osmY, vvhA, *δ*-vph), 0053-83 (pilV, pilW, tlh, hlyD, hlyIII, rtx), CIP 103381 (T6SS vasD), and 2232 (T6SS DUF877, T6SS DUF770) as well as ORF12 of CH-291 were used as reference sequences. Strains showing 90–100% sequence similarity to the specific reference sequence were defined as positive for the respective virulence-associated trait. WGS data were confirmed by PCR assays. VN-0392, VN-0507, and VN-0519 were PCR-negative for cps, T6SS DUF770 and pilW, respectively. WGS data showed primer mismatches*.

** *No verification of the WGS data by PCR assays*.

*** *Identical to ATCC 51183*.

For genes encoding hemolytic and cytolytic proteins, we chose homologs of *vvhA, tlh*, δ*-vph, hlyIII*, and *osmY*. The *vvhA* gene of *V. vulnificus* encodes a potent cytolytic hemolysin whose role in pathogenicity has been under debate (Jones and Oliver, 2009; Lee et al., 2013). The gene is present in clinical and environmental *V. vulnificus* strains and is used for species identification (Campbell and Wright, 2003). Similarly, a *tlh* homolog encoding a putative thermolabile hemolysin is found in many *Vibrio* species. Its role in pathogenicity is unclear (Zhang and Austin, 2005) and it is used in *V. parahaemolyticus* for species identification (Jones et al., 2014). WGS data indicated the presence of the two genes in all genomes and PCR assays for gene homologs of *vvhA* and *tlh* were positive in the 20 *V. navarrensis* strains of this study, which indicates that these genes might also be suitable for identification of this species (Table 4).

A gene encoding a putative hemolysin III family protein (HlyIII) with a size of 214 amino acids is annotated in the WGS data of all *V. navarrensis* strains (except VN-0507). Due to nucleotide sequence variations, PCR amplicons of this gene were not obtained for all strains (data not shown). In case of *V. vulnificus*, a homolog of the HlyIII protein was investigated in more detail (Chen et al., 2004). As a *hlyIII* mutant of *V. vulnificus* exhibited attenuated virulence in a mouse model compared with the wild-type strain, a role of HlyIII in virulence was suggested (Chen et al., 2004; Zhang and Austin, 2005). Another thermostable hemolysin, δ-VPH, with unclear role in pathogenicity has been found in *V. parahaemolyticus* and *V. cholerae* (Zhang and Austin, 2005). In contrast to *tlh, vvhA*, and *hlyIII*, the putative δ*-vph* gene was only detected in strains from marine environments (seawater strains and blue mussel strain) and in the two human pathogenic strains (WGS data). The *osmY* gene homolog encoding a putative hemolysin (accession KGK22069) with a domain for attachment to phospholipid membranes was present in all strains (Table 4).

WGS data of all strains showed the presence of a *hlyD* gene encoding a hemolysin D protein and an *rtx* gene encoding a repeats-in-toxin protein. HlyD proteins are involved in transport of hemolysins through the bacterial inner membrane (Pimenta et al., 2005; Linhartová et al., 2010), while secreted RTX proteins mostly exhibit pore-forming activity visible as hemolytic halo surrounding bacterial colonies on blood agar (Linhartová et al., 2010).

Jores et al. cloned a 15.6 kbp DNA fragment of *V. navarrensis* biotype *pommerensis* CH-291 into the plasmid pVH that upon introduction into *E. coli* strain DH5α conferred hemolytic properties. DNA hybridization experiments of the whole fragment were positive only with strains of the biotype *pommerensis* and were suggested to be specific for the biotype. The hemolytic properties were found on two neighboring regions of the 15.6 kbp fragment, each containing more than one open reading frame (ORF) (Jores et al., 2003). ORF12, the largest ORF conferring hemolytic properties, was only present in four strains (CH-271, CH-280, CH-291, VN-0516; Table 4). The significance of this region for identification of a subpopulation of *V. navarrensis* strains requires the study of more strains.

Type IV pilins of Gram-negative bacteria play various roles in pathogenicity (Giltner et al., 2012). In toxigenic *V. cholerae*, a type IV pilus is a major virulence factor that functions as an essential colonization factor and acts as cholera toxin phage receptor (Karaolis et al., 1998; Rivera et al., 2001). Two genes, *pilW* and *pilV*, coding for type IV pilus assembly or pilus biosynthesis proteins were present in most strains. The *pilV* gene was absent in three out of the 20 strains (WGS and PCR), whereas the *pilW* gene was found in all strains, although in one strain (VN-0519), a PCR to confirm the gene failed. A gene coding for a putative protein of capsule biosynthesis (designated as *cps*) was detected in all strains (Table 4).

Type VI secretion systems (T6SS) have attracted attention, as they play important roles in virulence of a number of Gram-negative bacteria by translocating effector proteins into eukaryotic cells. Recently, T6SS have also been shown to transport proteins into prokaryotic cells showing bactericidal activity against competitors (Ho et al., 2014). Three genes of putative T6SS proteins were tested for presence in *V. navarrensis* strains. Two of the genes encode proteins associated with the baseplate containing domains of unknown functions (T6SS DUF877 and T6SS DUF770) and one gene encodes a VasD protein homolog which is a lipoprotein tethered to the outer membrane. Interestingly, six of eight seawater strains were negative for these genes indicating that the T6SS is not present in these environmental strains. In contrast, the mussel strain as well as the reference strain CIP 103381 from sewage and all veterinary strains harbor the T6SS (Table 4). The WGS analysis confirmed the PCR results. WGS data of the two human pathogenic strains 0053-83 and 08-2462 revealed the absence of the three selected genes and indicate the lack of the T6SS in these strains.

In summary, no clear discrimination based on virulence-associated factors was observed between the strains, as most of the investigated genes were present in all strains. Some genes encoding virulence-associated traits may be useful for further analysis of strains from different origins. Candidate genes identified in this study are the δ*-vph* gene and genes encoding components of the T6SS and the hemolytic activity encoding region of biotype *pommerensis* strains. However, evidence if some of these genes contribute to a pathogenic potential do require additional research. It is feasible that discrimination of environmental and potentially pathogenic strains requires the identification of allelic variants of specific genes as it is the case for clinical strains of *V. vulnificus* (Jones and Oliver, 2009).

## Conclusion

This study was initiated by a recent publication about *V. navarrensis* strains recovered from human specimens. The strains originated from diverse human sources (blood, wound, ear, stool) suggesting that this species is a human pathogen. The veterinary isolates of this study were isolated from animals intended for food production in farms of the German state Saxony that has no border to marine environments. The strains were recovered after abortions from placentas and some strains were isolated directly from inner organs of the aborted fetuses. A pathogenic potential of these isolates seems likely. However, it cannot be excluded that the strains were purely commensals, as no further investigations regarding pathogenicity were performed. The animal source of the strains is unusual, as *Vibrio* bacteria are mostly found in marine environments and are commonly associated with marine organisms. The uptake through feed of marine origin (seafeed) was discussed; however, no satisfying explanation for the occurrence of *Vibrio* strains in domestic animals was found. Cases of human vibriosis result either through contact to seawater or by uptake of contaminated seafood. The occurrence of possibly pathogenic *Vibrio* strains in mammalian hosts intended for food production is of great interest, as it could indicate a so far unrecognized source of *Vibrio* infections.

The isolation of *V. navarrensis* from domestic animals after miscarriages and from diseased humans suggests a pathogenic potential of these bacteria and could mean that this species is a so far unrealized zoonotic agent. The “One Health” concept acknowledges that human, animal, and environmental health are linked. Further research is necessary to identify reservoirs, sources, and ways of transmission of this species to determine a possible role as zoonotic agent.

## Author contributions

KS, NB, KT, and ES designed the study. KS, CK, and NB performed the experiments. KS, CK, NB, JH, KT, and ES analyzed the data. KS, JH, and ES prepared the tables and figures, wrote the manuscript. All authors edited the manuscript.

### Conflict of interest statement

The authors declare that the research was conducted in the absence of any commercial or financial relationships that could be construed as a potential conflict of interest.
