# Supplementary material for: Diversity of Vibrio navarrensis Revealed by Genomic Comparison: Veterinary Isolates Are Related to Strains Associated with Human Illness and Sewage Isolates While Seawater Strains Are More Distant
Source: Front Microbiol. 2017 Sep 6;8:1717. doi: 10.3389/fmicb.2017.01717 (PMC5592226; doi:10.3389/fmicb.2017.01717)
Supplement: Supplementary file 1 [file Image1.PDF]

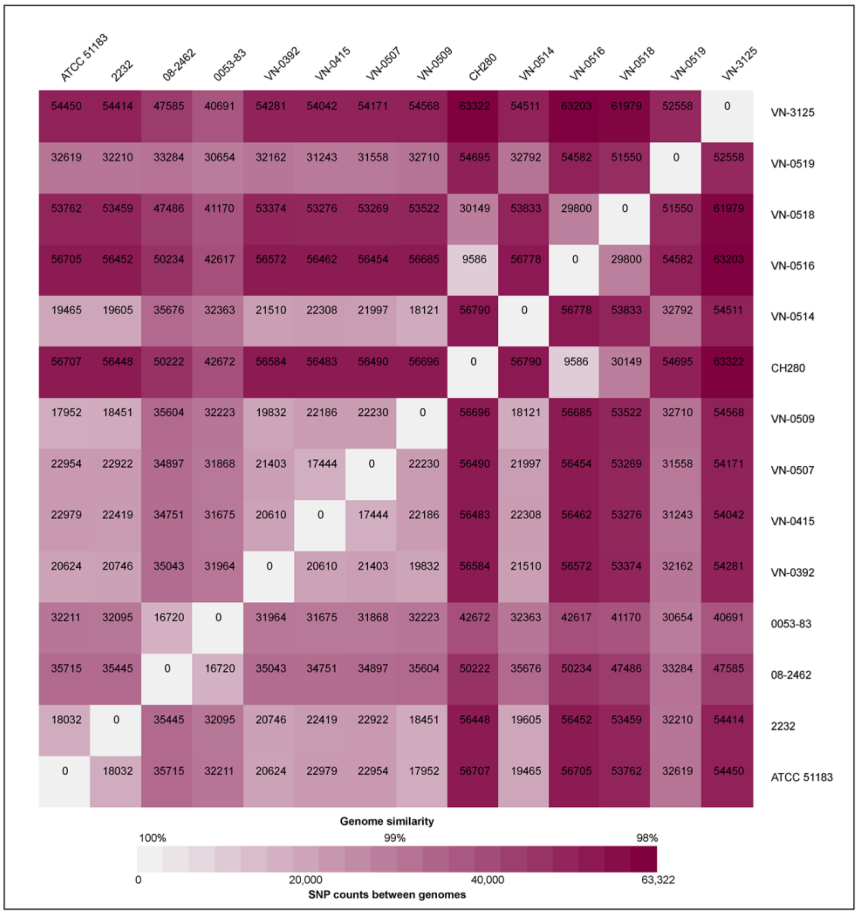

Figure S1. SNP distance matrix of 14 *V. navarrensis* strains analyzed in this study. The strains originated from different sources: veterinary/Germany (VN-0392, VN-0415, VN-0507, VN-0509, VN-0514), environmental/Germany (CH-280, VN-0516, VN-0518, VN-0519, VN-3125), human/U.S. (0053-83, 08-2462), and environmental/Spain (ATCC 51183 (identical to CIP 103381), 2232). SNP difference between each pair of strains was calculated by using CSI Phylogeny 1.4 (see Material and Methods) and is displayed numerically and graphically as a purple square with a color intensity proportional to the number of SNP differences, but inversely proportional to the percentage genome similarity.
