# Supplementary material for: Diversity of Vibrio navarrensis Revealed by Genomic Comparison: Veterinary Isolates Are Related to Strains Associated with Human Illness and Sewage Isolates While Seawater Strains Are More Distant
Source: Front Microbiol. 2017 Sep 6;8:1717. doi: 10.3389/fmicb.2017.01717 (PMC5592226; doi:10.3389/fmicb.2017.01717)
Supplement: Supplementary file 2 [file Table1.PDF]

Table S1. PCR primers used for *rpoB* sequence determination, MLSA, and genotyping of virulence-associated genes. Primer sequences were derived from whole genome shotgun contigs of the *V. navarrensis* strains 0053-83 (JMCF01000000), 08-2462 (JMCI01000000), and ATCC 51183 (JMCG01000000) or from the partial ORF12 sequence of *V. navarrensis* CH-291 (AJ314791).

Target gene	Primer	Sequence (5‘ to 3‘)	Amplicon size (bp)	Reference / Accession
rpoB	1110F	GTAGAAATCTACCGCATGATG	984	Tarr et al., 2007
	CM32b	CGGAACGGCCTGACGTTGCAT		
	1661F*	TTYATGGAYCARAACAACCC	-	Tarr et al., 2007
	1783R*	GGACCTTYAGGNGTTTCGAT		
gyrB	VigyrBF	GAAGGTGGTATTCAAGCGTT	570**	http://pubmlst.org/vibrio/info/Vibrio_primers.pdf
	VigyrBR	CGGTCATGATGATGATGTTGT		
pyrH	VipyrHdgF	CCCTAAACCAGCGTATCAACGTATTC	501**	http://pubmlst.org/vibrio/info/Vibrio_primers.pdf
	VipyrHdgR	CGGATWGGCATTTTGTGGTCACGWGC		
recA	VirecAF	TGCGCTAGGTCAAATTGAAA	462**	http://pubmlst.org/vibrio/info/Vibrio_primers.pdf
	VirecAdgR	GTTTCWGGGTTACCRAACATYACACC		
atpA	Vi_atpAdg_F	ATCGGTGACCGTCARACWGGTAAAAC	489**	http://pubmlst.org/vibrio/info/Vibrio_primers.pdf
	Vi_atpAdg_R	ATACCTGGGTCAACCGCTGG		
cps	Nav-cps-F	TGATGGTGGTGCTGGTAATG	349	JMCF01000086
	Nav-cps-R	TGTTCCAGACGTTCAAGCC		
T6SS *DUF877*	Nav-T6-DUF877-F	TCTCAGCAGAACAGCTAACC	345	JMCG01000001
	Nav-T6-DUF877-R	ACTCATCCCAAGTACAAGCC		
T6SS *DUF770*	Nav-T6-DUF770-F	GTCAAAACACCGAAGCTCTC	358	JMCG01000001
	Nav-T6-DUF770-R	CCAATCTTCAGTTCATCAGCC		
T6SS *vasD*	Nav-T6-vasD-F	GTTGTAGTGCGGCGAATATG	198	JMCG01000001
	Nav-T6-vasD-R	TATCTGGCCCGAGAACTTCAC		
tlh	Nav-tlh-F	GCACCGGAAACTCTCATAAC	425	JMCF01000074
	Nav-tlh-R	TTTCTCTTGGGCGATGTATTC		
osmY	Nav-osmY-F	ACACCAGAACCACCAAAGAG	301	JMCI01000041
	Nav-osmY-R	GCCAACAATGCGGATTTGAC		
vvhA	Nav-vvhA-F	GCCGATTTACATCACCAGCTC	438	JMCG01000001
	Nav-vvhA-R	GCCCCCACTTTACCATTCAC		

Table continued				
**Target gene**	**Primer**	**Sequence (5‘ to 3‘)**	**Amplicon size (bp)**	**Reference / Accession**
δ-vph	Nav-vph-F	ACTTACATCGCTTTATGCCC	407	JMCI01000055
	Nav-vph-R	CGAGTGCTGATAGTAACTTCC		
hlyD	Nav-hlyD-F	AAGAACAAGCGATTCCACTC	430	JMCF01000099
	Nav-hlyD-R	AAACGACTATCCACAATGCC		
pilV	Nav-pilV-F	GAACAAAAAGCCGACTACGC	213	JMCI01000074
	Nav-pilV-R	TTTCAATGCCCCGGAGAGAG		
pilW	Nav-pilW-F	AGTAACCAACAATGCCGACCC	189	JMCF01000057
	Nav-pilW-R	TTGCCATTTTCGCCGACCAG		
ORF12	Vnav-pom-HemF	GAATACTTCCCACGGTGAAAC	547	AJ314791
	Vnav-pom-HemR	CAAACCAATCACGCTCAATG		

* Sequencing primer.
** Amplicon length for MLSA after trimming.
