# Supplementary material for: Diversity of Vibrio navarrensis Revealed by Genomic Comparison: Veterinary Isolates Are Related to Strains Associated with Human Illness and Sewage Isolates While Seawater Strains Are More Distant
Source: Front Microbiol. 2017 Sep 6;8:1717. doi: 10.3389/fmicb.2017.01717 (PMC5592226; doi:10.3389/fmicb.2017.01717)
Supplement: Supplementary file 3 [file Table2.PDF]

Table S2. Prophage analysis in *V. navarrensis* genomes of veterinary and environmental strains.

Predicted prophage	Length (kb)	Completeness	Score	Predicted CDS	Related phage (Accession No.)	GC content (%)
VN-0392_PP01	31.3	Questionable	80	21	*Vibrio* phage VP882 (NC_009016)	46.83
VN-0392_PP02	11.7	Intact	120	16	Enterobacteria phage HK630 (NC_019723)	51.46
VN-0392_PP03	15.2	Incomplete	20	22	*Shigella* phage SfII (NC_021857)	46.50
VN-0415_PP01	7.6	Incomplete	10	7	*Bacillus* phage G (NC_023719)	49.61
VN-0415_PP02	7.9	Incomplete	10	8	*Bacillus* phage G (NC_023719)	47.96
VN-0415_PP03	8.9	Incomplete	10	11	*Acanthamoeba* mimivirus (NC_014649)	49.37
VN-0415_PP04	7.3	Incomplete	10	9	*Burkholderia* phage phi1026b (NC_005284)	51.92
VN-0415_PP05	10.3	Incomplete	20	11	*P. dulcis* phage (NC_021858)	50.04
VN-0415_PP06	7.2	Incomplete	20	7	*Natrialba* phage PhiCh1 (NC_004084)	47.18
VN-0415_PP07	7.9	Incomplete	30	8	*Acanthamoeba* mimivirus (NC_014649)	47.07
VN-0507_PP01	7.3	Incomplete	10	9	*Burkholderia* phage phi1026b (NC_005284)	51.92
VN-0507_PP02	7.6	Incomplete	10	7	*Bacillus* phage G (NC_023719)	49.60
VN-0507_PP03	14.3	Incomplete	20	8	*Planktothrix* phage PaV-LD (NC_016564)	46.51
VN-0507_PP04	26.5	Incomplete	30	14	*P. dulcis* phage (NC_021858)	44.52
VN-0507_PP05	8.9	Incomplete	10	11	*Acanthamoeba* mimivirus (NC_014649)	49.44
VN-0507_PP06	7.2	Incomplete	20	7	*E. siliculosus* virus 1 (NC_002687)	47.16
VN-0507_PP07	7.9	Incomplete	10	8	*Bacillus* phage G (NC_023719)	48.02
VN-0509_PP01	10.8	Incomplete	20	21	*Shigella* phage SfIV (NC_022749)	46.99
VN-0509_PP02	20.6	Intact	150	20	Enterobacteria phage HK629 (NC_019711)	52.55
VN-0514_PP01	7.6	Incomplete	10	7	*Bacillus* phage G (NC_023719)	49.49
VN-0514_PP02	19.9	Incomplete	50	8	*Bacillus* phage vB_BanS-Tsamsa (NC_023007)	52.46
VN-0514_PP03	7.2	Incomplete	20	7	*E. siliculosus* virus 1 (NC_002687)	47.27
VN-0514_PP04	8.9	Incomplete	10	11	*Acanthamoeba* mimivirus (NC_014649)	49.42
VN-0514_PP05	13.6	Incomplete	20	7	*Planktothrix* phage PaV-LD (NC_016564)	46.18
VN-0514_PP06	7.9	Incomplete	10	8	*Bacillus* phage G (NC_023719)	47.95
VN-0514_PP07	10.3	Incomplete	20	11	*P. dulcis* phage (NC_021858)	50.09
VN-0514_PP08	7.3	Incomplete	10	8	*Burkholderia* phage phi1026b (NC_005284)	51.89
VN-0514_PP09	8.1	Incomplete	10	9	Enterobacteria phage phi92 (NC_023693)	44.60

Table continued						
**Predicted prophage**	**Length (kb)**	**Completeness**	**Score**	**Predicted CDS**	**Related phage (Accession No.)**	**GC content (%)**
VN-0516_PP01	11.5	Intact	136	14	*Vibrio* phage VCY phi (NC_016162)	43.96
VN-0518_PP01	11.0	Questionable	83	10	*Vibrio* phage fs2 (NC_001956)	44.42
VN-0518_PP02	20.8	Incomplete	50	16	*P. dulcis* phage (NC_021858)	52.13
VN-0519_PP01	23.3	Incomplete	30	21	*Mannheimia* phage vB_MhS_1152AP2 (NC_028956)	48.67
VN-0519_PP02	41.3	Questionable	70	43	Enterobacteria phage VT2phi_272 (NC_028656)	48.83
VN-0519_PP03	46.6	Incomplete	60	20	*P. dulcis* phage (NC_021858)	49.10
VN-0519_PP04	37.0	Intact	110	46	*Vibrio* phage 12B12 (NC_021070)	50.00
VN-3125_PP01	10.9	Intact	136	15	*Vibrio* phage VCY phi (NC_016162)	45.17
VN-3125_PP02	20.6	Questionable	20	23	*Shigella* phage SfII (NC_021857)	46.42
VN-3125_PP03	20.4	Intact	140	20	Enterobacteria phage HK630 (NC_019723)	52.81
VN-3125_PP04	38.6	Intact	150	52	*Vibrio* phage 8 (NC_022747)	48.64
CH-280_PP01	14.2	Incomplete	20	7	*Planktothrix* phage PaV-LD (NC_016564)	46.63
CH-280_PP02	7.6	Incomplete	10	7	*Bacillus* phage G (NC_023719)	49.38
CH-280_PP03	7.9	Incomplete	10	8	*Bacillus* phage G (NC_023719)	47.78
CH-280_PP04	7.3	Incomplete	10	9	*Burkholderia* phage phi1026b (NC_005284)	52.12
CH-280_PP05	8.9	Incomplete	10	11	*Acanthamoeba* mimivirus (NC_014649)	49.21
CH-280_PP06	10.3	Incomplete	20	11	*P. dulcis* phage (NC_021858)	49.79
CH-280_PP07	10.1	Questionable	70	14	*Bacillus* phage G (NC_023719)	50.53
